# Analysis of Macular Drusen and Blood Test Results in 945 *Macaca fascicularis*

**DOI:** 10.1371/journal.pone.0164899

**Published:** 2016-10-24

**Authors:** Koji M. Nishiguchi, Yu Yokoyama, Yusuke Fujii, Kosuke Fujita, Yusuke Tomiyama, Ryo Kawasaki, Toshinori Furukawa, Fumiko Ono, Nobuhiro Shimozawa, Mutsumi Togo, Michihiro Suzuki, Toru Nakazawa

**Affiliations:** 1 Department of Advanced Ophthalmic Medicine, Tohoku University Graduate School of Medicine, Sendai, Japan; 2 Department of Ophthalmology, Tohoku University Graduate School of Medicine, Sendai, Japan; 3 Department of Retinal Disease Control, Tohoku University Graduate School of Medicine, Sendai, Japan; 4 Department of Public Health, Yamagata University Graduate School of Medical Science, Yamagata, Japan; 5 Kurashiki University of Science and the Arts, Department of Comparative Animal Science, Kurashiki, Japan; 6 Chiba Institute of Science, Faculties of Risk and Crisis Management, Choshi, Japan; 7 Tsukuba Primate Research Center, National Institute of Biomedical Innovation, Health and Nutrition, Tsukuba, Japan; 8 The Corporation for Production and Research of Laboratory Primates, Tsukuba, Japan; University of Melbourne, AUSTRALIA

## Abstract

Age-dependent formation of macular drusen caused by the focal accumulation of extracellular deposits beneath the retinal pigment epithelium precede the development of age-related macular degeneration (AMD), one of the leading causes of blindness worldwide. It is established that inflammation contributes to the pathogenesis of drusen and AMD. However, development of a preemptive therapeutic strategy targeting macular drusen and AMD has been impeded by the lack of relevant animal models because most laboratory animals lack macula, an anatomic feature present only in humans and a subset of monkeys. Reportedly, macular drusen and macular degeneration develop in monkeys in an age-dependent manner. In this study, we analyzed blood test results from 945 *Macaca fascicularis*, 317 with and 628 without drusen. First, a trend test for drusen frequency (the Cochran–Armitage test) was applied to the quartile data for each parameter. We selected variables with an increasing or decreasing trend with higher quartiles at P < 0.05, to which multivariate logistic regression analysis was applied. This revealed a positive association of age (odds ratio [OR]: 1.10 per year, 95% confidence interval [CI]: 1.07–1.12) and white blood cell count (OR: 1.01 per 1 × 10^3^/μl, 95% CI: 1.00–1.01) with drusen. When the monkeys were divided by age, the association between drusen and white blood cell count was only evident in younger monkeys (OR: 1.01 per 1 × 10^3^/μl, 95% CI: 1.00–1.02). In conclusion, age and white blood cell count may be associated with drusen development in *M*. *fascicularis*. Systemic inflammation may contribute to drusen formation in monkeys.

## Introduction

Age-related macular degeneration (AMD) is the leading cause of blindness in elderly residents of industrialized countries [1]. The disease is classifiable into “wet” and “dry” forms based on distinct clinical features. In wet AMD, rapid visual loss is caused by macular choroidal neovascularization. Dry AMD entails a slower degeneration of the retinal pigment epithelium, choroid, and surrounding extracellular matrix in the macular area. Before diverging into these two distinct forms of the disease, both conditions are preceded by the accumulation of extracellular aggregates, termed drusen, between the retinal pigment epithelium and Bruch’s membrane.

Unlike wet AMD, for which effective treatments exist [2–4], therapeutic options are lacking for dry AMD, partly because of a lack of appropriate animal models that recapitulate the complex clinical features of the condition. To date, mice have commonly been used to study isolated aspects of AMD because of their availability and suitability for genome manipulation. These efforts have greatly enhanced our understanding of the pathology of AMD, but insufficiently. Unfortunately, a fundamental limitation hampers the use of mice as animal models of macular degeneration: they lack a macula, a unique anatomic feature present only in humans and a subset of monkeys. Therefore, when studying macular disease in animals, monkeys with a macula are preferred over other species. Previous studies have reported that macular drusen are prevalent in various monkeys worldwide, including *Macaca mulatta* and *Macaca fascicularis*, which are frequently used in biomedical research [5–7]. In *M*. *fascicularis*, a family with early onset drusen inherited in an autosomal-dominant manner has been reported [8], along with monkeys with drusen possibly inherited in a non-Mendelian manner [7] within the same colony.

During the past decade, several large-scale genetic studies targeting patients with AMD have identified disease-associated *genes* and single-nucleotide variants [9]. Interestingly, many AMD-associated *genes* were found to encode members of complement pathways, including *CFH* [10–12], *C2/CFB* [13], *C3* [14], *CFI* [15–17], and *C9* [15, 18]. Complement pathways are ubiquitous inflammatory systems activated against pathogens and inflammatory stimuli in multiple organs throughout the body. Members of these pathways are often secreted into the bloodstream as soluble factors. Consequently, the alteration of complement pathways can affect systemic inflammatory biomarkers in the blood. For example, increased white blood cell count [19] and C-reactive protein level [20] are associated with AMD, which is consistent with the genetic findings. In monkeys, local ocular involvement of complement pathways has been detected by immunohistochemistry and proteome analysis using ocular samples from *M*. *fascicularis* with drusen [21, 22]. The same study group also conducted proteome analysis of plasma samples from *M*. *fascicularis* with and without drusen. They identified ApoE as a potential biomarker of the disease [23]. However, each study examined only a few monkeys. Another study of *M*. *mulatta* implicated genetic risk *genes* shared between monkeys with drusen and human patients with AMD[24].

In this study, we compared the results of standard blood tests in a large colony of *M*. *fascicularis* with and without drusen to identify systemic biomarkers of drusen and ascertain whether these markers overlap with those reported in humans.

## Materials and Methods

### Animals

We examined 1,174 *M*. *fascicularis* reared at Tsukuba Primate Research Center at the National Institutes of Biomedical Innovation, Health and Nutrition (NIBIOHN), Tsukuba, Japan [25]. The monkeys ranged in age from 1–38 years. They were housed in an indoor environment where artificial lighting was used for 12 h each day. The animals were fed 70 g of commercial food (CMK-2; CLEA Japan, Inc., Tokyo, Japan) and 100 g of apples daily. Tap water was supplied *ad libitum*. Every morning their health status (e.g., viability, appetite, coat appearance) was monitored. The monkeys were provided with toys, branches, and music as a part of efforts to improve their enrichment. The maintenance of animals was conducted according to the rules for animal care of the Tsukuba Primate Research Center for the care and use of, and biohazard countermeasures related to, laboratory animals. All animal experiments were conducted in accordance with the guidelines for animal experiments of the NIBIOHN and with the *Guide for the Care and Use of Laboratory Animals* of the National Institutes of Health (Bethesda, MD, USA). The research protocol was approved by the ethics committee at the Tsukuba Primate Research Center.

### Fundus photography and blood test

Approximately 20 min before examining the ocular fundi, a mixture of tropicamide and phenylephrine hydrochloride was instilled into both eyes of each animal to dilate the pupils. Then, the monkeys were anesthetized with an intramuscular injection of ketamine (10.0 mg/kg). Fundus photographs were taken with an ophthalmoscope camera (Kowa RC-2; Kowa Co. Ltd., Tokyo, Japan). A monkey was categorized as having drusen if one or more round yellowish spots with the characteristic appearance of drusen, regardless of their size or location, were identified in either eye or both eyes in a fundus photo of the posterior pole centered on the macula that encompassed ~23° vertically and ~19° horizontally. The quality of photos of nine monkeys was too poor to determine the presence or absence of drusen; thus, these monkeys were excluded from further analysis. All images were assessed by an experienced ophthalmologist and a veterinarian specializing in ophthalmology to ascertain the presence or absence of drusen. In most cases, the two assessors agreed on the interpretation of the photos (Cohen's kappa index value: 0.962). However, when there were disagreements, the fundus photos were reviewed together and decisions were made after a discussion.

The body weight of each animal was measured. A blood sample was obtained from the femoral vein. A proportion of the blood was subjected to hematologic analysis. Serum was isolated from the remainder to perform biochemical analysis. The blood testing was performed as a part of a routine health-monitoring program unrelated to the current project by technical staff at the Tsukuba Primate Research Center under the direction of a veterinarian.

### Statistical analysis

First, we evaluated the relationships among the parameters by calculating the Pearson correlation coefficient. For the pair of parameters that exhibited a high correlation coefficient (*r* > 0.600), one was excluded from further analysis. Then, continuous variables were divided into quartiles with the first quartile as the reference group, to which two statistical analyses were applied before selecting variables to be analyzed by logistic regression analysis. Odds ratios (ORs) were calculated with and without adjustment for age and sex (either age or sex for subgroup analysis). To assess the ORs for the second, third, and fourth quartiles with the first quartile as reference for each variable, logistic regression analysis was applied. Then, the Cochran–Armitage test was used to objectively assess the trend for drusen frequency using the quartile data. Variables that showed an increasing or decreasing trend (P < 0.050) with higher quartiles were further selected for logistic regression analysis to independently assess the effect of each of the selected variables. A multiple logistic regression model refined by stepwise procedures using the backward entry method was applied to estimate the risks of potential predictors, including age, sex, and selected blood parameters, for the development of macular drusen. The Akaike information criterion (AIC) was used to determine the variables to be added to or deleted from the model. Only the set of variables that minimized the AIC value was retained in the final model. We repeated the variable selection using the P-value (< 0.150) to determine the best subset of variables for the model; the models also yielded similar results as that generated using the AIC. In addition, the collinearity of parameters retained in the final model was assessed using the variance inflation factor (VIF). All VIF values were less than 10.0, which meant that there was no collinearity in the model.

Sub-population analysis was also carried out. The monkeys were divided into two groups, i.e., males (*n* = 232) and females (*n* = 713; subgroup analysis 1), and younger (1–6 years; *n* = 477) and older (older than 6 years; *n* = 468; subgroup analysis 2), and an identical analysis workflow as that applied to the monkeys as a single group was employed. R software (version 3.2.2; R Foundation for Statistical Computing, Vienna, Austria) was used for all calculations.

## Results

**Fig 1:**
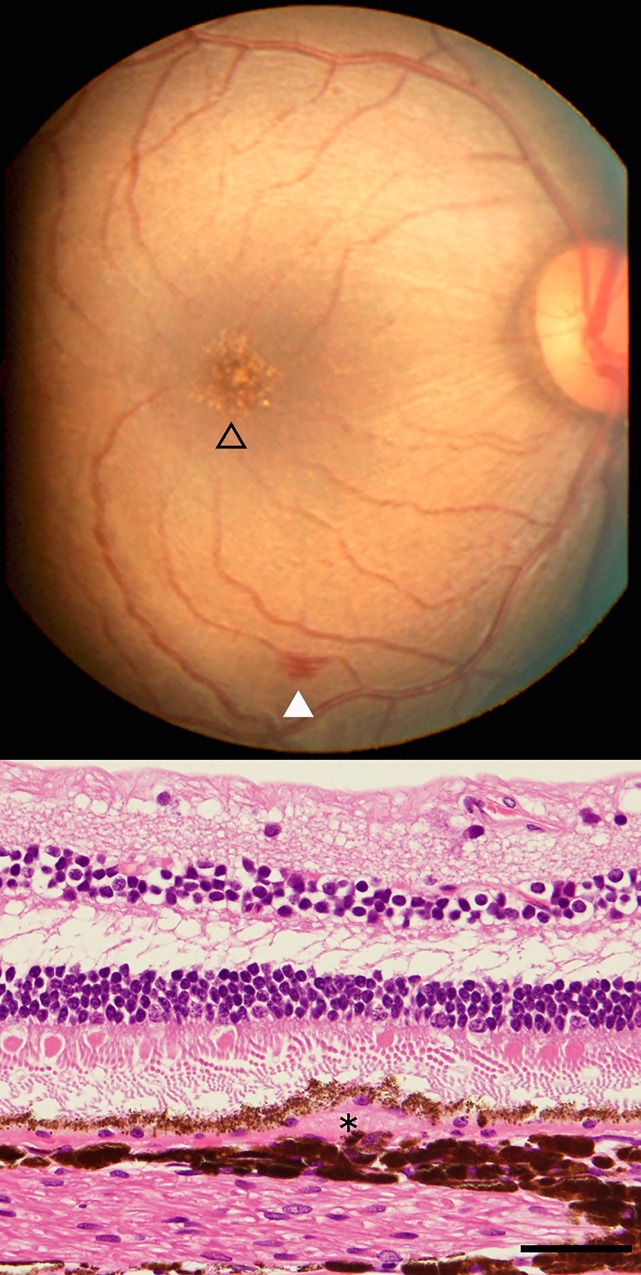
Fundus of a monkey eye with drusen. Fundus photograph of a 21-year-old male *Macaca fascicularis* with drusen (open triangle; upper panel). Note retinal hemorrhage is seen inferiorly (filled triangle). Histologic section of an eye with drusen from another 34-year-old monkey. Note an accumulation of extracellular material underneath the retinal pigment epithelium consistent with drusen formation (*).

Fundus photographs were taken in 1,174 *M*. *fascicularis* between 2011 and 2013 (Fig 1). The blood tests included a complete blood cell count and a standard biochemical analysis. Of the 1,174 monkeys, only those with a complete dataset, including biologic data and all basic blood data, and with discernable fundus photos were analyzed further. This analysis included 945 monkeys, comprising 317 with and 628 without drusen. The biologic distribution of each blood test parameter is presented in S1 Fig. Comparison of the biologic and basic blood test data between monkeys with and without drusen are presented in Table 1. Applying a Mann–Whitney *U*-test to each parameter revealed several statistically significantly different parameters between the two groups.

**Table 1:** Basic biologic and hematologic data for monkeys with and without drusen.

	Unit	Total	Drusen +	Drusen -	*P*-value*
**Number**		945	317	628	-
**Sex**	**%Male**	24.6	21.5	26.1	0.115*
**Weight**	**kg**	3.38 ± 1.19	3.67 ± 1.27	3.23 ± 1.13	< 0.001
**Age**	**years**	8.34 ± 5.87	10.46 ± 6.35	7.28 ± 5.31	< 0.001
**WBC**	**x10**^**3**^**/**μ**l**	6.52 ± 2.67	6.74 ± 2.76	6.42 ± 2.62	0.034
**RBC**	**x10**^**4**^**/**μ**l**	558 ± 64	564 ± 66	555 ± 62	0.090
**Hemoglobin**	**g/dl**	11.0 ± 1.0	11.1 ± 1.1	11.0 ± 1.0	0.102
**Platelet**	**x10**^**5/**^μ**l**	3.21 ± 0.90	3.30 ± 0.96	3.17 ± 0.87	0.051
**Protein**	**g/dl**	7.05 ± 0.51	7.08 ± 0.53	7.03 ± 0.50	0.045
**Albumin**	**g/dl**	4.11 ± 0.34	4.06 ± 0.33	4.14 ± 0.35	< 0.001
**BUN**	**mg/dl**	17.5 ± 5.4	17.1 ± 5.8	17.7 ± 5.2	0.016
**Glucose**	**mg/dl**	45.5 ± 20.0	47.9 ± 25.0	44.3 ± 16.7	0.054
**Cholesterol**	**mg/dl**	111 ± 30	115 ± 30	108 ± 30	< 0.001
**Tryglyceride**	**mg/dl**	45.4 ± 45.3	50.0 ± 51.5	43.2 ± 41.7	0.034
**Phosphate**	**mg/dl**	4.52 ± 1.47	4.31 ± 1.31	4.62 ± 1.54	0.002
**Calcium**	**mg/dl**	9.21 ± 0.56	9.19 ± 0.70	9.22 ± 0.48	0.864
**GOT**	**IU/l**	53.7 ± 19.0	53.7 ± 18.2	53.7 ± 19. 5	0.990
**GPT**	**IU/l**	74.6 ± 57.7	78.1 ± 62.0	72. 7 ± 55.4	0.245
**CRP**	**mg/dl**	0.111 ± 0.219	0.127 ± 0.239	0.103 ± 0.208	0.017

*Except for sex, which was evaluated using the chi-squared test, the Mann–Whitney *U*-test was used to test for differences between monkeys with and without drusen. Data represents the mean ± one standard deviation. WBC, white blood cells; RBC, red blood cells; BUN, blood urea nitrogen; GOT, glutamate oxaloactetate transaminase; GPT, guanine phosphoribosyl transferase; CRP, C-reactive protein.

To test if any of the variables were related, we calculated the Pearson correlation coefficient (*r*) between all the variables (S1 Table). Age and weight and red blood cell count and hemoglobin exhibited correlations (*r* > 0.600). At this point, the data for weight and hemoglobin were excluded from the analysis. Then, quartile data for each variable were analyzed to detect significant associations with the frequency of drusen (Table 2). When logistic regression analysis was applied to the quartile data, ORs were consistently increased for the three higher quartiles for age and white blood cell count. Then we assessed if the quartile data showed a significantly increasing or decreasing trend (Table 2). We selected seven variables with an increasing or decreasing trend at P < 0.050 and applied multivariate logistic regression analysis. As a result, only two factors remained (Table 3). These were age (OR: 1.10 per year, 95% confidence interval [CI]: 1.03–1.18; P = 0.004) and white blood cell count (OR: 1.01 per 1 × 10^3^/μl, 95% CI: 1.00–1.02; P = 0.179).

**Table 2:** Quartile analyses and trend tests in 945 monkeys.

Variables	Quartiles	crude OR (95%CI)	adjusted OR (95%CI)	*P*-value*	*P*-value†
Age	Q1	1.00	1.00	-	
	Q2	2.26 (1.41–3.61)	2.24 (1.40–3.58)	< 0.001	
	Q3	2.21 (1.40–3.49)	2.18 (1.38–3.45)	< 0.001	
	Q4	5.22 (3.37–8.08)	5.13 (3.30–7.97)	< 0.001	<0.001
WBC	Q1	1.00	1.00	-	
	Q2	1.70 (1.14–2.55)	1.63 (1.08–2.48)	0.020	
	Q3	1.78 (1.20–2.66)	1.68 (1.11–2.53)	0.013	
	Q4	1.66 (1.11–2.48)	1.65 (1.09–2.49)	0.018	0.018
RBC	Q1	1.00	1.00	-	
	Q2	1.49 (1.01–2.19)	1.50 (1.01–2.23)	0.047	
	Q3	1.13 (0.76–1.68)	1.12 (0.74–1.68)	0.591	
	Q4	1.51 (1.03–2.23)	1.25 (0.83–1.89)	0.278	0.128
Platelet	Q1	1.00	1.00	-	
	Q2	0.94 (0.64–1.40)	1.04 (0.70–1.57)	0.834	
	Q3	1.00 (0.68–1.48)	1.07 (0.72–1.61)	0.726	
	Q4	1.34 (0.91–1.96)	1.42 (0.95–2.11)	0.087	0.122
Protein	Q1	1.00	1.00	-	
	Q2	0.71 (0.46–1.08)	0.66 (0.43–1.02)	0.063	
	Q3	0.93 (0.63–1.37)	0.76 (0.51–1.14)	0.190	
	Q4	1.17 (0.79–1.72)	0.74 (0.49–1.13)	0.159	0.189
Albumin	Q1	1.00	1.00	-	
	Q2	0.82 (0.55–1.21)	1.01 (0.67–1.52)	0.965	
	Q3	0.58 (0.36–0.94)	0.77 (0.47–1.26)	0.302	
	Q4	0.51 (0.34–0.75)	0.77 (0.51–1.18)	0.236	<0.001
Glucose	Q1	1.00	1.00	-	
	Q2	0.98 (0.66–1.46)	0.94 (0.63–1.42)	0.776	
	Q3	1.07 (0.73–1.58)	0.99 (0.66–1.47)	0.948	
	Q4	1.37 (0.93–2.00)	0.98 (0.65–1.48)	0.933	0.088
BUN	Q1	1.00	1.00	-	
	Q2	0.69 (0.47–1.00)	0.75 (0.50–1.10)	0.142	
	Q3	0.74 (0.51–1.08)	0.86 (0.58–1.27)	0.448	
	Q4	0.61 (0.41–0.89)	0.78 (0.52–1.17)	0.233	0.022
Cholesterol	Q1	1.00	1.00	-	
	Q2	1.40 (0.94–2.09)	1.44 (0.96–2.18)	0.079	
	Q3	1.45 (0.98–2.16)	1.36 (0.91–2.05)	0.138	
	Q4	1.96 (1.33–2.91)	1.26 (0.82–1.92)	0.296	0.001
Triglyceride	Q1	1.00	1.00	-	
	Q2	0.77 (0.52–1.14)	0.77 (0.51–1.15)	0.199	
	Q3	1.10 (0.75–1.62)	0.97 (0.65–1.45)	0.877	
	Q4	1.25 (0.85–1.84)	0.80 (0.52–1.21)	0.290	0.081
Phosphate	Q1	1.00	1.00	-	
	Q2	1.00 (0.68–1.48)	1.17 (0.78–1.74)	0.450	
	Q3	1.09 (0.75–1.59)	1.53 (1.02–2.27)	0.038	
	Q4	0.51 (0.35–0.76)	0.88 (0.57–1.36)	0.553	0.003
Calcium	Q1	1.00	1.00	-	
	Q2	0.83 (0.55–1.24)	0.82 (0.54–1.24)	0.347	
	Q3	0.85 (0.57–1.26)	0.92 (0.61–1.39)	0.702	
	Q4	0.90 (0.61–1.32)	1.14 (0.76–1.70)	0.525	0.700
GOT	Q1	1.00	1.00	-	
	Q2	0.88 (0.60–1.29)	0.94 (0.63–1.41)	0.775	
	Q3	1.11 (0.75–1.64)	1.32 (0.88–1.99)	0.178	
	Q4	0.89 (0.60–1.31)	1.08 (0.72–1.63)	0.698	0.864
GPT	Q1	1.00	1.00	-	
	Q2	1.08 (0.73–1.59)	1.15 (0.77–1.71)	0.504	
	Q3	1.09 (0.74–1.61)	1.13 (0.75–1.69)	0.556	
	Q4	1.28 (0.87–1.89)	1.18 (0.79–1.76)	0.420	0.223
CRP	Q1	1.00	1.00	-	
	Q2	0.66 (0.42–1.02)	0.61 (0.39–0.96)	0.034	
	Q3	0.93 (0.63–1.38)	0.88 (0.58–1.32)	0.525	
	Q4	1.29 (0.86–1.93)	0.95 (0.62–1.46)	0.823	0.045

Odds ratios (ORs) for the second, third, and fourth quartiles with the first quartile as a reference are presented for each variable. Crude ORs and ORs adjusted for sex and age are displayed. Logistic regression analysis was applied to the second, third, and fourth quartiles with the first quartile as a reference, and P-values* are presented for each variable. The Cochran–Armitage test was used to test for trends (P-value: †) OR, odds ratio; CI, confidence interval.

**Table 3:** Multiple logistic regression analysis and associated factors for retinal drusen.

	adjusted OR(95%CI)	*P* value
Variables		
WBC (per 1x10^3^/μl)	1.01 (1.00–1.02)	0.179
Age (per 1 year)	1.10 (1.03–1.18)	0.004
Interaction term		
WBC×Age		0.818

WBC, white blood cell; OR, odds ratio; CI, confidence interval

A sub-population analysis was conducted to further investigate the data. First, sex-related differences were assessed, because reports suggest that AMD-associated factors may differ between human males and females [26, 27]. The monkeys were divided into males (*n* = 232) and females (*n* = 713). Quartile analysis followed by a trend test for drusen frequency (Cochran–Armitage test; S2 Table) was applied to each sex group following the protocol used to analyze all 945 monkeys. The quartile analysis showed an age-dependent increase in drusen development to be prominent in females but not in males. We then selected variables with an increasing or decreasing trend at P < 0.050 and applied multivariate logistic regression analysis. When stepwise multiple logistic regression analysis was applied, three factors and one factor remained in the final model for males and females, respectively (Table 4). In either sex, only age was statistically significant, with an OR of 1.07 per year (95% CI 1.02–1.13) for males and 1.1 per year (95% CI 1.07–1.13) for females. For males, red blood cell count and blood urea nitrogen remained in the final model, but they did not reach statistical significance. The VIF was ascertained to check for multicollinearity between the variables. All VIF values were less than 10.0, which meant that there was no collinearity in the model.

**Table 4:** Multiple logistic regression analysis and associated factors for retinal drusen in 232 male and 713 female monkeys.

Variables	Odds ratio (Unit)	95% CI	*P* value	VIF
**Male (N = 232)**				
Age	1.07 (per 1 year)	1.02–1.13	0.003	1.03
RBC	1.00 (per 1x10^4^/μl)	0.99–1.01	0.119	1.05
BUN	0.96 (per 1 mg/dl)	0.90–1.01	0.136	1.05
**Female (N = 713)**				
Age	1.10 (per 1 year)	1.07–1.13	<0.001	-

RBC, red blood cell; BUN, blood urea nitrogen; CI, confidence interval; VIF, variance inflation factor.

Next, the monkeys were divided into two groups based on age, i.e., the younger (1–6 years; *n* = 477) and older (7 years or older; *n* = 468) groups. This was to test if the etiology of drusen present in younger animals differed from those present in older animals, because drusen are almost never observed in young humans. A quartile analysis followed by a trend test for drusen frequency (Cochran–Armitage test; S3 Table) was applied to each of the two age groups. The quartile analysis showed the effect of the increase in white blood cell count on drusen frequency to be more prominent in young monkeys compared with older monkeys. We then selected variables with an increasing or decreasing trend at P < 0.050 and applied multivariate logistic regression analysis. When stepwise multiple logistic regression analysis was applied, three factors and one factor remained in the final model for younger and older monkeys, respectively (Table 5). In either group, age remained in the final formula. In both groups, age was statistically significant, with an OR of 1.3 (95% CI: 1.10–1.53) for younger monkeys and 1.08 (95% CI: 1.04–1.12) for older monkeys. For the younger monkeys, white blood cell count remained in the formula, with an OR of 1.01 (95% CI: 1.00–1.01), and showed statistical significance. In older monkeys, Albumin remained in the final model, but it did not reach statistical significance. The VIF was ascertained to check for multicollinearity between the variables. All VIF values were less than 10.0, which meant that there was no collinearity in the model.

**Table 5:** Multiple logistic regression analysis and associated factors for retinal drusen in younger and older monkeys.

Variables	Odds ratio (Unit)	95% CI	*P* value	VIF
**Aged six years or younger (N = 477)**				
Age	1.30 (per 1 year)	1.10–1.53	0.002	1.01
WBC	1.01 (per 1x10^3^/μl)	1.00–1.02	0.009	1.01
**Aged seven years or older (N = 468)**				
Age	1.08 (per 1 year)	1.04–1.12	< 0.001	1.02
Albumin	0.64 (per 1g/dl)	0.38–1.06	0.087	1.02

WBC, white blood cells; CI, confidence interval; VIF, variance inflation factor.

## Discussion

In this study, we investigated non-ocular factors associated with drusen by analyzing biologic data and blood test results from 945 *M*. *fascicularis*. To the best of our knowledge, this is the largest study to screen the fundus for drusen and analyze blood samples to assess systemic involvement in monkeys with ocular diseases.

As with humans [28], the development of drusen was associated most strongly with increasing age in these monkeys, consistent with previous reports [6, 29–31]. This was the case when all monkeys were assessed together or divided into male and female or young and old groups. Interestingly, age seemed to have the largest effect on young monkeys, with an OR (1.30) exceeding those of the other subgroups. In addition to age, an increased white blood cell count was also associated with drusen when all 945 monkeys were assessed together and when younger monkeys (6 years of age or younger) were selected for analysis.

An increased white blood cell count is linked to an elevated incidence of early AMD in humans [19]. It is particularly interesting that the association was evident in this study in younger monkeys and not in older monkeys, which implies that an age-related difference in the pathogenesis of drusen may exist at least in this species. In *M*. *fascicularis*, monkeys with early onset drusen inherited in an autosomal-dominant manner [8], and also monkeys with drusen inherited in a non-Mendelian manner, have been reported [7]. Unfortunately, information related to their pedigrees is unavailable. Nevertheless, because drusen are quite common in monkeys of various species, and were found in approximately 35% of the monkeys examined in this study, with an age-dependent increase in its prevalence even in older monkeys, it is likely that drusen are not inherited as a discrete early onset autosomal-dominant disease in most monkeys. These findings, which are consistent with the idea that systemic inflammation also underlies the formation of drusen in monkeys, are intriguing. The OR for white blood cell count in the logistic regression model appeared low (1.01 per 1 × 10^3^/μl). However, the white blood cell count was highly variable between the monkeys (1.2–11.9 × 10^3^/μl, a range for mean ± two standard deviations). This implies the differential importance of the count as a potential risk factor for drusen in these monkeys. The reason for this increase in white blood cell count is uncertain. It is possible that it reflects chronic infection by pathogens, such as *Chlamydia pneumonia* [32], that might affect the clinical course of AMD.

The main limitation of this study is the missing link between drusen formation and macular degeneration in monkeys. The phenotypic features of drusen in monkeys and humans are quite different. It seems that drusen can appear from a young age and are usually small, punctate, and concentrated around the fovea (see Fig 1 for typical macular drusen appearance) in monkeys, which differs from the typical profiles of drusen found in aged humans. In humans, an association between larger, “soft” drusen and the development of advanced AMD is established, whereas the pathogenicity of small, punctate, “hard” drusen is considered less significant. However, the molecular components of soft and hard drusen are not markedly different [33]. Furthermore, evidence suggests that small, punctate, hard drusen that resemble those found in monkeys may also precede the development of dry AMD in humans [34–38]. Therefore, taking into account all the discrepancies between drusen in humans and monkeys, there is little doubt that these monkeys are one of the best animal models of human macular drusen available. As the monkeys studied are from an inbred colony, some monkeys may be related to each other. This is another limitation of the study, because this indicates that the data from these monkeys are not likely to be independent.

In conclusion, this study analyzed basic blood tests, including complete blood count and blood chemistry, in a large colony of *M*. *fascicularis* with and without drusen. Our results show associations of age and white blood cell count with drusen development. Systemic inflammation may underlie drusen formation in monkeys as it does in humans, which further highlights the relevance of monkeys with drusen as potential models of early AMD.

## Supporting Information

S1 Fig The biological distribution of the blood test parameters. Y.O., year old; WBC, white blood cells; RBC, red blood cells; HGB, hemoglobin; PLT, platelet; ALB, albumin; BUN, blood urea nitrogen; GLU, glucose; TCHO, total cholesterol; TG, triglyceride; P, phosphate; Ca, calcium; GOT, glutamate oxaloactetate transaminase; TP, total protein; GPT, guanine phosphoribosyl transferase; CRP, C-reactive protein.
(DOCX)

S1 Table The correlation between numeric variables. Pearson correlation coefficient is displayed. Absolute r > 0.6 is highlighted in red.
(XLSX)

S2 Table Separate quartile analyses for male and female monkeys. Odds ratio for 2nd, 3rd, 4th quartile with 1st quartile as the reference is presented for each variable, separately for the male and female monkeys. Crude odds ratio and odds ratio adjusted for age are displayed. Logistic regression analysis was applied to the 2nd, 3rd, 4th quartile with the 1st quartile as reference and *P*-value* was presented for each variable. Cochran-Armitage test was applied to test for trend (*P*-value†) OR, odds ratio; CI, confidence interval.
(XLSX)

S3 Table Separate quartile analyses for younger and older monkeys. Odds ratio for 2nd, 3rd, 4th quartile with 1st quartile as the reference is presented for each variable, separately for the male and female monkeys. Crude odds ratio and odds ratio adjusted for sex are displayed. Logistic regression analysis was applied to the 2nd, 3rd, 4th quartile with the 1st quartile as reference and *P*-value* was presented for each variable. Cochran-Armitage test was applied to test for trend (*P*-value†) OR, odds ratio; CI, confidence interval. Aged 6 years or younger, N = 477; Older than 6 years, N = 468.
(XLSX)
