# Supplementary material for: Analysis of Macular Drusen and Blood Test Results in 945 Macaca fascicularis
Source: PLoS One. 2016 Oct 24;11(10):e0164899. doi: 10.1371/journal.pone.0164899 (PMC5077098; doi:10.1371/journal.pone.0164899)
Supplement: S1 Fig — Y.O., year old; WBC, white blood cells; RBC, red blood cells; HGB, hemoglobin; PLT, platelet; ALB, albumin; BUN, blood urea nitrogen; GLU, glucose; TCHO, total cholesterol; TG, triglyceride; P, phosphate; Ca, calcium; GOT, glutamate oxaloactetate transaminase; TP, total protein; GPT, guanine phosphoribosyl transferase; CRP, C-reactive protein. (DOCX) [file pone.0164899.s001.docx]

**Supplementary Figure 1. The biological distribution of the blood test parameters**

Y.O., year old; WBC, white blood cells; RBC, red blood cells; HGB, hemoglobin; PLT, platelet; ALB, albumin; BUN, blood urea nitrogen; GLU, glucose; TCHO, total cholesterol; TG, triglyceride; P, phosphate; Ca, calcium; GOT, glutamate oxaloactetate transaminase; TP, total protein; GPT, guanine phosphoribosyl transferase; CRP, C-reactive protein.
